# Material‐Induced Venosome‐Supported Bone Tubes

**DOI:** 10.1002/advs.201900844

**Published:** 2019-07-01

**Authors:** Baptiste Charbonnier, Aslan Baradaran, Daisuke Sato, Osama Alghamdi, Zishuai Zhang, Yu‐Ling Zhang, Uwe Gbureck, Mirko Gilardino, Edward Harvey, Nicholas Makhoul, Jake Barralet

**Affiliations:** ^1^ Department of Mechanical Engineering McGill University 817 Sherbrooke Street West Montreal H3A 0C3 Quebec Canada; ^2^ Experimental Surgery Division Department of Surgery Faculty of Medicine Montreal General Hospital 1650 Cedar Avenue Montreal H3G 1A4 Quebec Canada; ^3^ Department of Implant Dentistry Showa University Dental Hospital 2 Chome‐1‐1 Kitasenzoku Ota City Tokyo 145‐8515 Japan; ^4^ Division of Oral & Maxillofacial Surgery McGill University Montreal General Hospital 1650 Cedar Avenue Montreal H3G 1A4 Quebec Canada; ^5^ Faculty of Dentistry McGill University 3640, Strathcona Anatomy and Dentistry Building, University Street Montreal H3A 0C7 Quebec Canada; ^6^ Department for Functional Materials in Medicine and Dentistry University of Würzburg Pleicherwall 2 D‐97070 Würzburg Germany

**Keywords:** angiogenesis, axial vascularization, bioceramic, bioinorganic, material–host interactions, osteogenesis

## Abstract

The development of alternatives to vascular bone grafts, the current clinical standard for the surgical repair of large segmental bone defects still today represents an unmet medical need. The subcutaneous formation of transplantable bone has been successfully achieved in scaffolds axially perfused by an arteriovenous loop (AVL) and seeded with bone marrow stromal cells or loaded with inductive proteins. Although demonstrating clinical potential, AVL‐based approaches involve complex microsurgical techniques and thus are not in widespread use. In this study, 3D‐printed microporous bioceramics, loaded with autologous total bone marrow obtained by needle aspiration, are placed around and next to an unoperated femoral vein for 8 weeks to assess the effect of a central flow‐through vein on bone formation from marrow in a subcutaneous site. A greater volume of new bone tissue is observed in scaffolds perfused by a central vein compared with the nonperfused negative control. These analyses are confirmed and supplemented by calcified and decalcified histology. This is highly significant as it indicates that transplantable vascularized bone can be grown using dispensable vein and marrow tissue only. This is the first report illustrating the capacity of an intrinsic vascularization by a single vein to support ectopic bone formation from untreated marrow.

## 1 Introduction

Microsurgery (suturing nerves and vessels ≈1 mm in diameter) transformed reconstructive surgery allowing a procedure known as free flap or free tissue transfer. This then allowed replantation of digits and ears and then famously, harvesting of a nonessential bone (e.g. ilium or fibula) with its feeding artery and draining vein, shaping and transplanting it.^1^ This procedure remains, after more than half a century, the standard autograft reconstruction for patients who lose mandibles or long bone segments through cancer or trauma. It is nonideal; there is poor anatomic fit and hospital recovery is extended.^2^

Scientists then attempted to revascularize necrotic and nonvital bone:^3^ in a landmark study, ligated arteries, ligated arteriovenous bundles (AVB), and veins anastomosed to arteries (arteriovenous loop, AVL) were shown to form new vessels, and the clinical potential of the AVB was established.^4^ Erol and Spira investigated various AVL configurations and reported the ability of all to generate new capillary beds.^5^ The concept of generating tissues de novo from existing vessels soon followed and Fisher and Yang reported the formation of a vascularized ear and penis in pig models using the gastroepiploic vessels and omentum.^6^ The AVL is less preferred over AVB because it requires harvesting of a vein and anastomosis of a vein to an artery, which is time consuming and may fail,^4^ but is reported to generate more new vessels than the AVB.^4^, ^7^, ^8^, ^9^ There are two types of AVB, one in which the bundle is ligated, and another known as “flow‐through” where the scaffold is placed around an arteriovenous bundle in which side branches are trimmed and ligated to provide two unbranched straight vessels. While this AVB method avoids sacrifice of an artery through ligation, an artery still needs to be cut and reattached if the newly created tissue is to be transplanted. Tanaka et al. reported the first systematic comparison of AVL and both types of AVB to vascularize a biomaterial intended to create an engineered vascularized dermis.^4^ They found the least vascularization in the flow‐through AVB group, and attributed what little there was to inflammation and surgical injury. Since then there have been scores of studies of attempts to engineer skin, bone, and other tissues using all three kinds of vascular induction, collectively known as intrinsic vascularization.^10^ Despite extensive research, intrinsic vascularization approaches have been trialed clinically but are not in common usage.^11^, ^12^ Further details concerning the currently reported approaches to engineer vascularized scaffolds for bone regeneration are provided in a recent review by Barabaschi et al.^13^

Since the discovery of these techniques 40 years ago, there has never been any study examining the capacity of a vein alone to develop a new vasculature when a material is placed around it. This may be because the original intent of intrinsic vascularization was to supply arterial blood and it was thought that dilation of the vein and shear stresses caused by exposing it to arterial blood pressure was a main driver of luminal sprouting.^14^, ^15^ Unlike a pressurized artery, segments of vein are surgically dispensable and harvesting causes minimal functional impact to the patient.

Attempts to grow bone have combined all three intrinsic vascularization methods with various scaffold materials, cell types, and inductive proteins; mainly bone morphogenetic protein, stem cells, and autograft.^16^ Marrow aspirate is a tissue that can be harvested without significant harm, under local anesthetic, and in large quantitates.^17^ It is usually used clinically in combination with other graft materials and is a source of stem, endothelial, osteogenic, and hemopoietic cells, but is alone considered inadequate for significant bone formation.^18^ A review of the literature indicated that there have only been two reports using unmodified marrow aspirate supported in a biomaterial with axial perfusion.^19^, ^20^ One compared an AVL perfusion with direct implantation of a marrow‐loaded tricalcium phosphate but reported no difference in bone formation with or without perfusion,^20^ and the other perfused marrow loaded in a polymer ceramic composite with an AVB, but no mention of bone formation was reported, even at 10 weeks.^19^

Here, we then investigate for the first time whether a vein alone can undergo luminal sprouting following circumferential placement of a bioceramic material. Having established this property, we sought to determine if the material‐induced venosome could support significant bone growth from untreated bone marrow aspirate supported in the bioceramic, in the absence of an intrinsic feeding arterial vessel.

## 2 Results

### 2.1 Implant Characterization

**Figure 1:**
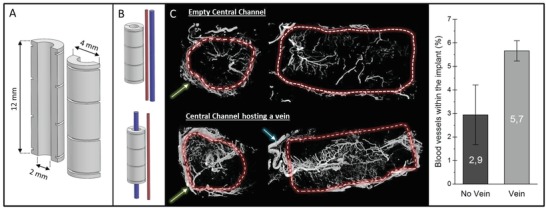
A) CAD design of cylindrical implant, B) schematic of the control placement next to femoral arteriovenous bundle and the experimental group with a single femoral vein placed axially within implant. C) Micro‐CT cross‐sections of decalcified implants perfused with MicroFil showing the blood vessels inside and outside the implant (green arrow: fibrous capsule; blue arrow: feeding vein) and quantification of the relative volume of blood vessels inside the implant.

**Figure 2:**
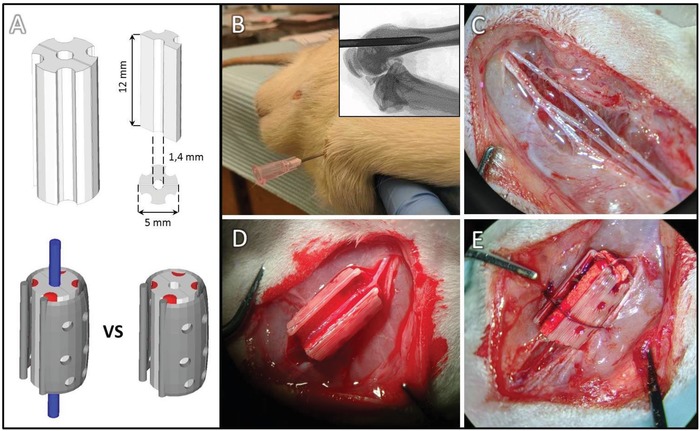
A) Marrow retaining scaffold design and dimensions and positioning of vessels with perforated plastic retainer. B) Marrow aspiration procedure and C–E) scaffold assembly in plastic retainer.

Microporous bioceramics, 12 mm long, were 3D printed as split tubes either with a cylindrical or cross‐shaped profile (**Figures** **1** and **2**), and were 43.5% porous monetite converted from brushite made by powder printing tricalcium phosphate (TCP) with phosphoric acid, as reported previously.^21^ Scaffold composition was monetite (80.2 wt%) and unreacted α‐ and β‐TCP (1.8 and 18 wt%) and they had bimodal porosity distributions 10 µm > 57.1 vol% > 1 µm and 1 µm > 29.8 vol% > 100 nm.

### 2.2 Explant Analyses

Pilot experiments to determine if an isolated vein could be induced to sprout by the circumferential placement of a biomaterial confirmed that the central vein did develop a neo‐venosome that extended throughout the monetite matrix in 3/3 samples. The control was extrinsically vascularized but at a much lower vessel density (Figure 1C, 2.9 ± 1.3% vs 5.7 ± 0.4%).

Having established that veins could be induced to sprout to form new venosomes, we sought to determine if marrow‐loaded scaffolds would form bone more readily when intrinsically vascularized with an induced venosome, compared with random extrinsic capillary ingrowth. Four longitudinal grooves were designed to accommodate the marrow and a plastic clip held the scaffold halves closed (Figure 2A) and shielded the marrow laden grooves so that this marrow tissue was not displaced during implantation (Figure 2B–E).

**Figure 3:**
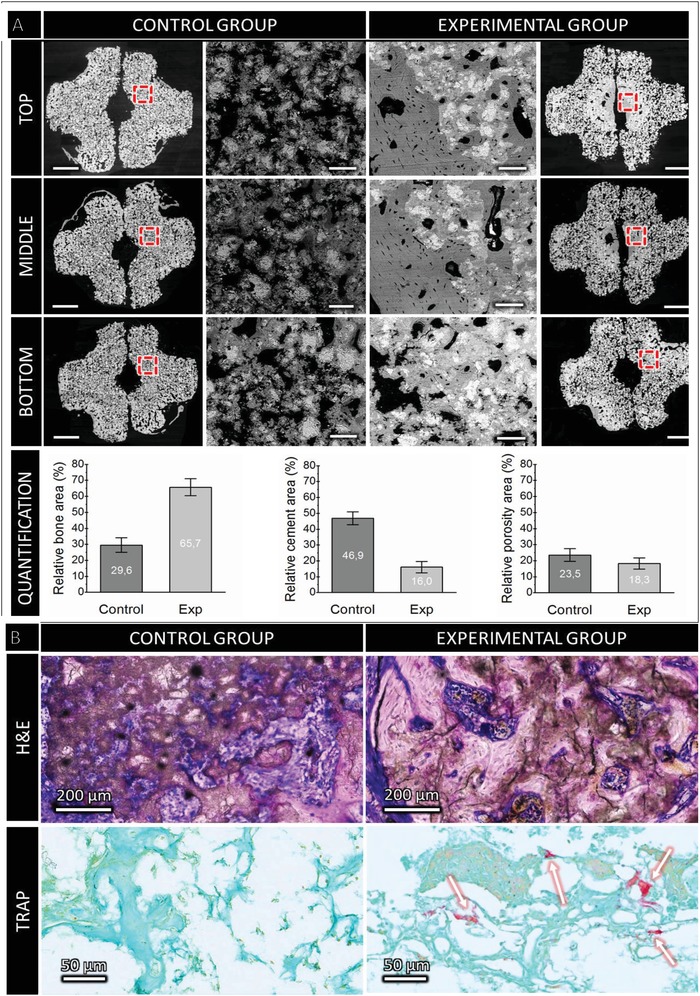
A) Backscattered SEM axial sections from the top, middle, and bottom of scaffolds with high magnification regions. Scale bars: 1 mm and 100 µm, for the low and high magnification, respectively. Significant (*P* < 0.0001) differences between bone volume and residual cement areas beween groups were observed. B) Microstructure of construct (H&E stained axial cross‐section), left, without intrinsic venous perfusion, new bone (pink) on surface of cement (dark brown and white) with interstitial fibrous tissue; right with induced venosome, significant and contiguous bone entombing residual cement. Bone was visible inside the implant of the vein group (e.g., H&E staining). TRAP positive staining detected only in sample with induced venosome (white arrows).

**Figure 4:**
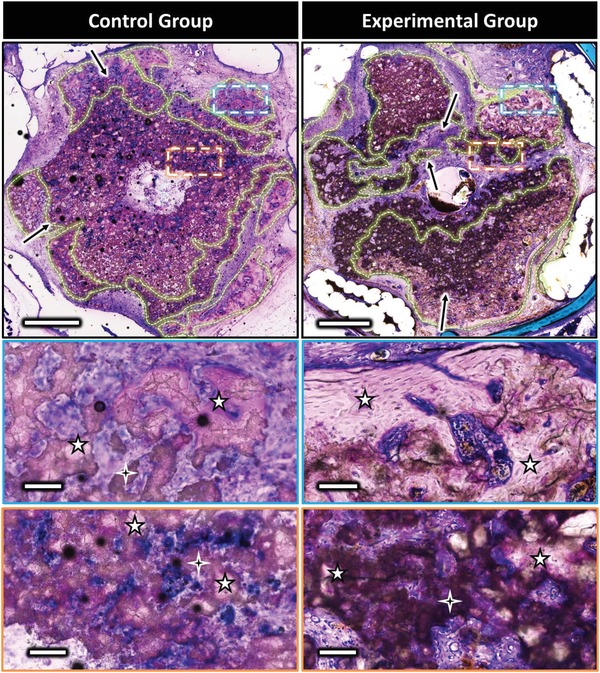
Representative mineralized axial sections from middle region of the control (left) and the experimental (right) scaffolds at low and high magnification (basic fuchsin and methylene blue). Zones illustrating the ceramic biodegradation are highlighted by the black arrows. Lamellar bone could be observed in the zones delineated by dotted lines, representing high magnification images shown in insets. The 4 and 5 branch stars indicate biocement and bone, respectively. Scale bars on low and high magnification represent 1 mm and 100 µm, respectively. Sections of replicate sample are shown in Figure S2 in the Supporting Information.

After 8 weeks of implantation, the implants were excised and characterized by micro‐CT and scanning electron microscopy (SEM). **Figure** **3** shows axial sections comparing top, middle, and bottom regions of the scaffold. It is apparent that there was some bone formation in the outer region of the control; in some cases bone spicules bridged the outer corners of the grooves. The experimental group had much higher resorption of the scaffold as can be determined visually by the greater proportion of black (porosity), and the central region was often comprised mainly of bone. Backscattered SEM of the same axial sections shown by micro‐CT better differentiated bone from residual ceramic and allowed quantification of bone and cement area (Figure 3). Bone formation was more than doubled when perfused by the venosome and resorption of the graft was tripled (*P* < 0.0001). Tartrate resistant acid phosphatase (TRAP) positive staining confirmed active remodeling by osteoclasts in scaffolds with the venosome but was not detected in the extrinsically vascularized controls. Mineralized sections confirmed bone formation within the original scaffold volume (Figures 3 and **4**; Figure S2, Supporting Information).

Bone could be observed outside and inside the bioceramics in both control and experimental groups, with the presence of mature bone bridges spanning the grooves. However, this phenomenon was qualitatively observed as more frequent when the scaffolds were axially perfused, with bone bridges in some cases extended along the entire ceramic length (Figure S1, Supporting Information). Formation of bone inside the scaffold central channel was only observed for the experimental group samples (Figure 3, SEM images). Quantitative SEM analyses demonstrated that bone formation was higher for the experimental group than for the control (Figure 3, *P* < 0.0001). The area of bone formation with a central vein was 66% with a 99.9% confidence interval of 61–71%, and without the vein the bone volume was 30% with a 99.9% confidence interval of 26–34% (*N* = 5 samples, triplicate measurements).

Mineralized histology confirmed electron microscopy and tomography observations. The bone formation was remarkable in that it was mainly contained within the original scaffold volume. In the vein‐free scaffolds, significant degradation was evident in the outer ≈500 µm, whereas the venously perfused scaffold was degraded throughout the matrix (Figure 4, black arrows) with pores from about 20–200 µm colonized by bone (Figure 4). In the non‐perfused control, mature bone with clear osteocytes was observed mainly in the outer 300–400 µm of the scaffold, and sporadically as small bone islands of 40–80 µm in diameter within the remaining bioceramic but not in the central channel (Figure 4). In the induced venosome group, bone was found throughout the scaffold and particularly near the central vein. Regions where bone was observed are shown with dotted lines to illustrate the differences (Figure 4, green zones).

**Figure 5:**
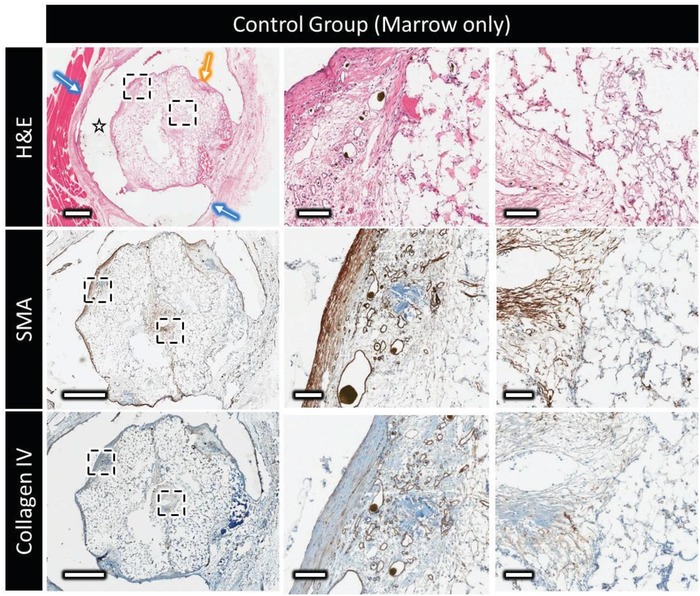
Decalcified histology and immunohistochemistry of the control marrow construct, showing top to bottom: H&E at low and high magnification showing the distribution and organization of the extracellular matrix outside and within the scaffold (star = polymer clip, blue arrow = thick vascular capsule surrounding the clip, and yellow arrow = thin vascular capsule surrounding the implant); the expression of α‐smooth muscle actin (brown); type‐IV collagen distribution (in brown). Scale bars on low and high magnification represent 1 mm and 100 µm, respectively.

**Figure 6:**
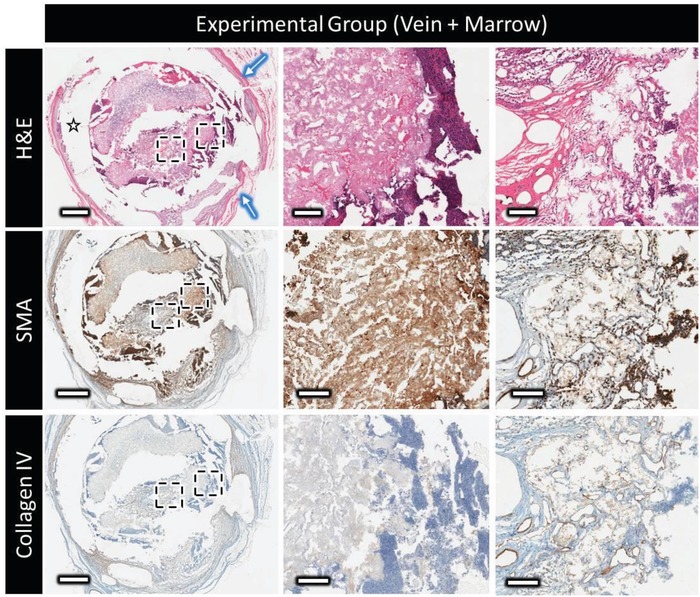
Decalcified histology and immunohistochemistry of the experimental group (marrow + vein perfusion), showing top to bottom: H&E at low and high magnification showing the distribution and organization of the extracellular matrix outside and within the scaffold (star = polymer clip and blue arrow = thick vascular capsule surrounding the clip); the expression of α‐smooth muscle actin (displayed in brown); type‐IV collagen distribution (in brown). Scale bars on low and high magnification represent 1 mm and 100 µm, respectively. Contrast absence of collagen IV staining inside the scaffold with the nonperfused sample in Figure 5.

Hematoxylin and eosin (H&E) staining of decalcified samples (**Figures** **5** and **6**) revealed different distributions and organizations of the extracellular matrix for the control and the experimental group samples. A thick vascularized fibrous capsule (300–600 µm) was always observed around the polymer clip (Figures 5 and 6, blue arrow), and a thin fibrous capsule (40–100 µm) observed surrounding the bioceramic implant of the control group (Figure 5, orange arrow) was absent in the experimental group. Extracellular matrix (ECM) could be found in scattered islands within the bioceramic walls and forming layers parallel to the inner surface in the central channel for the control group. In the vascularized scaffolds, ECM distribution was more homogeneous inside the bioceramic and seemed to be organized around the central blood vessel.

For the implants without axial vascularization, α‐smooth muscle actin (SMA) could be observed (Figures 5 and 6, SMA) in the thin vascular fibrous capsule around the bioceramic implant, around larger blood vessels, and had a layered organization in the central channel of the bioceramic. Many isolated myofibroblasts (20–50 µm) were evident inside the porosity of the bioceramic. In the venosome perfused marrow‐loaded samples, SMA positive tissue was found in axially perfused bioceramics in high concentrations in the external grooves, inside the bioceramic volume, and in the periphery of the central channel a few hundred micrometers thick.

In the nonperfused control group, collagen type IV staining indicative of blood vessel basement membrane indicated that blood vessels were mainly in the fibrous capsule surrounding the implant, and colonized the scaffold porosity, eventually reaching the central channel. A large majority of the blood vessels inside the scaffold were of small diameter (mainly less than 50 µm), and the vessels present in the central channel did not appear to extend onto the central region of the scaffold. On the contrary, when axially perfused, a dense vascular network composed of large‐ (>500 µm), medium‐ (250–500 µm), and small‐ (<150 µm) diameter blood vessels could be observed around the main vein in the implant central channel. The larger vessels, often parallel to the main vein, divided into smaller ones that did extend into the ceramic inner walls. Interestingly, no basement membrane was stained inside the scaffold volume, despite red blood cells being clearly visible in H&E stained sections. This is consistent with the lack of basement membrane found in most blood vessels inside bone, indicating that these blood vessels were potentially templating bone formation.^22^

SMA staining provided additional information as to the nature of the formed blood vessels, some displaying a thick SMA‐stained layer (≈10 µm) indicative of arterioles and others presenting a thinner SMA‐stained layer (>2 µm) were morphologically characteristic of venules, however no obvious zones containing exclusively one type of the other were observed.

## 3 Discussion

While ectopic bone formation using marrow aspirate and marrow derived cells loaded in scaffolds has been reported^20^ (**Table** **1**), no difference with or without vascularization was observed and very little bone was formed. In another study, no bone was mentioned as having been formed.^19^ The monetite used in this study has much higher solubility that tricalcium phosphate used previously^20^ (Solubility product constant, *K*_sp_ ≈ 10^−6.5^ and 10^−28.9^, respectively)^23^ and the ability of this material to resorb and allow blood vessel infiltration and subsequent bone formation may have been a factor in this striking difference.

**Table 1:** Summary of literature reporting preclinical bone formation in marrow or MSC‐loaded scaffolds implanted ectopically (AVL‐arteriovenous loop, AVB, arteriovenous bundle). A complete review may be found in ref. 16

Implant	Cells	Bioactive substance	Species	Intrinsic vascularization mode	Implantation	Implantation site	Bone formation	Ref.
Titanium mesh	Bone marrow cells 20 × 10^6^ per implant	None	Rat	None	Up to 6 weeks	Subcutaneous	9 ± 6%	37
BCP	Rat BMSCs	None	Immunodeficient mouse	None	Up to 10 weeks	Subcutaneous	22 ± 3.6%	38
BCP	Sheep BMSCs	None	Immunodeficient mouse	None	Up to 24 weeks	Subcutaneous	Up to 8.4% after 8 weeks	39
β‐TCP	Human bone marrow concentrate	BMP 2	Immunodeficient mouse	None	Up to 4 weeks	Subcutaneous	10.2 ± 3.3%	40
BCP	Human BMSCs 4.0 × 10^6^ per implant	None	Immunodeficient mouse	None	Up to 8 weeks	Subcutaneous	15.9 ± 4.0%	41
HA	Expanded sheep BMSCs 0.5–1.5 × 10^6^ per implant	none	Immunodeficient mouse	None	Up to 8 weeks	Subcutaneous	19.8 ± 2.5%	42
β‐TCP	Bone marrow aspirate	None	Sheep	With or without AVL	Up to 6 months	Intramuscular	Without AVL: 23.7 ± 0.8% with AVL: 36.5 ± 2.6%	20
HA, Si‐TCP, decellularized bone	BMSCs	BMP‐2	Rat	AVL	Up to 12 weeks	Subcutaneous	Up to 21.6 ± 3.7%	43
Demineralized bone matrix + membrane	Endothelial progenitor cells per Osteoblasts	None	Rat	AVB	Up to 12 weeks	Subcutaneous	Up to 18.17 ± 0.5%	44
β‐TCP	BMSCs ≈5.5 × 10^5^ per implant	None	Rabbit	With or without AVL	Up to 8 weeks	Subcutaneous	Without AVL : 26.6 ± 3.5% with AVL : 42.8 ± 5.9%	31
HA, collagen, polylactic acid	Bone marrow		Rabbit	AVB	Up to 10 weeks	Intramuscular	Not quantified	19
β‐TCP	BMSCs	None	Rabbit	AVB	4 weeks	Intramuscular	Implant wrapped in periosteum = 14.82 ± 3.0% no bone without periosteum	45
HA, silica gel	Autologous blood		Sheep	AVL	Up to 18 weeks	Subcutaneous	Up to 1.8 ± 2.1%	46

Mostly bone marrow stromal cells (BMSCs), seeded within scaffolds at numbers in the order of 10^6^ (refer to examples in Table 1), have been widely studied. Although BMSCs have demonstrated a real potential to form new bone in vitro and within scaffolds, their mechanism of action in vivo is still not certain. Indeed, there is no reported evidence that in vivo outcomes (e.g., bone volume, density, maturity) are any better than when using total bone marrow aspirate, although marrow's ability to form bone is dependent on age, fat content, and site of harvest.

Given that BMSCs are thought to make up only a maximum of 0.001% of the total marrow cell population, this seems to suggest several possibilities: i) only very few BMSCs are required for bone regeneration, and ii) other marrow cells, ECM, and cytokines^24^, ^25^, ^26^, ^27^ could also contribute to bone regeneration in vivo either alone or in concert with BMSC. Furthermore, there is a growing body of evidence to suggest that BMSCs are very hypoxia resistant^28^ which challenges the assumption that vascularization is essential for BMSC survival.

Previous work to initiate the ectopic formation of bone tissue generated only from 9% to 26.6% after 6–8 weeks (Table 1). Our avascular control induced bone at levels comparable with the literature range but this was significantly higher (66 ± 6%, Figure 2) when the scaffolds were axially perfused by a vein. The rapid formation of a significant volume of mature bone perfused by a single neo‐venosome, fed by a single large diameter vessel, indicates that the creation of potentially transplantable vascularized tissue from dispensable vein and marrow tissue is feasible. We are uncertain as to why placing a material around a vein caused such significant luminal sprouting however it has been observed that loosely fitting external stents caused sprouting in arterialized pressurized vein grafts,^29^, ^30^ an effect that was, like AVL sprouting, attributed to shear and dilation of the thin walled vein subjected to pressures an order of magnitude higher than encountered physiologically. It would appear though that material placement and likely the ensuing inflammation might also be a driver for vein sprouting. Comparison with the literature suggests that venous perfusion is at least as efficient as AVL to induce ectopic bone formation within a marrow laden monetite scaffold.^20^, ^31^ The fast colonization of the scaffold by a new and dense neo‐venous network,^21^ enabled by monetite's relatively high solubility, and its anastomosis to arterial capillaries in the external fibrous capsule allowed the formation of a neo‐osteal tube perfused by a single flow through femoral vein. Ischemic skin flaps, in which either an artery or a vein was left attached to the ischemic region only avoided necrosis if the vein but not the artery was left intact, an approach the authors termed “superdrainage.”^32^ We posit that our induced venous network that develops de novo over a few weeks sprouting through the cement and connecting with external capillaries acts similarly to sustain bone tissue. This new approach that exploits the rapid material‐induced remodeling of the vein and marrow's apparent efficacy of producing significant bone when vascularized removes a regenerative roadblock offering a practical and less invasive route to growing new bone without recourse to biologics or cultured cells.

## 4 Conclusion

A first step toward the subcutaneous development of transplantable vascularized large bone volumes with a very limited use of surgery (i.e., only femoral vascular bundle dissection) and use of easily harvestable tissues (vein and marrow) was reported in this study. Subcutaneous bone formation within 66.0% of the initial 3D‐printed implant volume was successfully achieved for the first time by venous perfusion. This supports the possibility of future clinical potential of venous axial perfusion for the field of bone and potentially other tissues' regeneration.

## 5 Experimental Section

*Implant Design, Manufacturing, and Characterization*: The implants were designed using Alibre design Xpress 10.0 CAD software. As illustrated in Figures 1A and 2A, implants were designed in two halves (12 mm high) that, when assembled, created a central channel in which a vein could be hosted. To evaluate the ability of a single vein to create a vascularized network, cylindrical implants were designed (Figure 1A, pilot study). To investigate the ability of the vein to support the formation of a new bone tissue in presence of bone marrow, cross‐shaped implants were designed with concave zones to retain this viscous fluid. To maintain in place the 2 implant halves, either they were sutured or a macroporous (12 mm pore diameter) sheath was devised (Figure 2A), also helping preserve the marrow aspirate location during implantation.

Calcium phosphate scaffolds were produced by additive manufacturing according to a reactive 3D‐printing technique co‐developed by the authors.^33^, ^34^ In short, a reaction between tricalcium phosphate powders (α‐ and β‐TCP, Ca_3_(PO_4_)_2_) and diluted phosphoric acid (H_3_PO_4_) allowed for the area selective binding of the powder grains. After printing, samples were soaked in 20% phosphoric acid for 60 s, washed, and sterilized by autoclaving.^33^, ^34^, ^35^ A Fortus 400mc 3D printer (Stratasys, USA) allowed for the production of the sheath, using food‐grade, sterilizable, and certified biocompatible (ISO 10993 USP Class VI) acrylonitrile butadiene styrene (ABS, ABSM30i).

X‐ray diffraction patterns of the printed implant were recorded with a Siemens D5005 diffractometer (Siemens, Karlsruhe, Germany). A step size of 0.02° was used to measure from 20° to 40° 2θ range with a total measuring time of 3 s per step. Phases were identified and quantified (Rietveld Refinement analysis) using TOPAS 2.0 software (Bruker AXS, Karlsruhe, Germany) combined with the International Centre for Diffraction Data patterns serving as reference for alpha‐TCP, beta‐TCP, brushite, and monetite. Scaffold architecture was investigated by microtomography X (SkyScan 1172; SkyScan Kontich, Belgium) equipped with a 0.5 mm aluminum filter at a resolution of 12 µm. The microstructure of the implant was investigated using scanning electron microscopy (SEM, Hitachi S‐4700 FE‐SEM; Tokyo, Japan) at an accelerating voltage of 20 kV. The porosity and pore‐size distribution of the 3D‐printed implants were determined by Hg porosimetry (PASCAL 140/440, Porotec GmbH, Hofheim, Germany).

*Experiment Design and Surgical Procedures—Pilot Study*: A proof of concept to investigate the potential of a single vein, perfusing the implant to generate a new vascular network, was performed. In short, 3D‐printed monetite tubes were implanted around (*N* = 3) the femoral vein or next to the femoral vascular bundle (*N* = 3) of Wistar rats. After 4 weeks on implantation, animals were sacrificed and injected with a contrast agent (MicroFil) allowing the imaging by micro‐CT of the vascular network. The density of blood vessel within the scaffold was determined after by analysis of the micro‐CT data, after decalcification of the implants in EDTA (Figure 1), as described previously.^21^

*Experiment Design and Surgical Procedures—Experiment Design*: The animal study was performed with 16 male Wistar rats (400–500 g, retired breeders) after approval from McGill University Animal Care Committee (UACC, #7662). Animals were randomly assigned to the control and experimental group (*N* = 9 per group), comprising animals implanted with 3D‐printed ceramic scaffold soaked with autologous bone marrow with and without axial perfusion by the femoral vein, respectively.

*Experiment Design and Surgical Procedures—Surgical Methods*: The surgical procedure is illustrated in Figure 2. Animals were administered a mixture of carprofen and buprenorphine 30 min prior to the surgery for analgesia and anesthetized with isoflurane. Isotonic fluids were administered subcutaneously (0.2–0.5 mL per 10 g body weight) to maintain proper hydration throughout the surgery.

Bone marrow was harvested from a femur according to a minimally invasive technique to harvest bone marrow that was adapted from Ordodi et al.^36^ and kept with 1% Heparin solution on ice during the operation until seeding on the scaffold (Figure 2B). In brief, a flexion of the rat limb and a small incision (<1 mm) performed on rat knee allowed for exposing distal femoral articular surface. A 18 g needle was aligned with the femoral shaft, and then was inserted inside the bone following the medullary canal and aiming toward the greater trochanter. Around 1 mL of marrow was aspirated, and then the needle was removed and the incision sutured with a single stitch.

Scaffold subcutaneous implantation took place on the opposite femoral site. Beginning from the medial side of the knee, a vertical incision of skin was performed, and the fatty and muscle tissue were carefully dissected. Femoral vessels were exposed and dissected between the inguinal ligaments proximally and the bifurcation of the saphenous and popliteal vessels distally. Femoral vein was gently isolated from artery and nerve, as shown in Figure 2C. After being soaked with ≈300 µL autologous marrow, the first half of the scaffold and the ABS sheath were transferred in the surgical site and the vein positioned in the central channel (Figure 2D). Scaffold's second half was carefully slid over the first half, and the system was sutured together with 5‐0 Prolene nonabsorbable sutures including the underlying muscle to prevent any movement (Figure 2E). To further secure the scaffold, the overlying facia was placed over and sutured with Monocryl absorbable thread. Finally wound was closed with the same absorbable suture. During the whole surgery, special attention was given not to compromise blood flow either, within the scaffold (vein) or in the femoral artery. For the control group, the two halves of the bone‐marrow‐soaked implant were inserted in the ABS sheath, and the system was simply placed next to the femoral vascular bundle and then secured as previously described.

*Experiment Design and Surgical Procedures—Retrieval and Analysis of the Explants*: After 8 weeks of implantation, animals were sacrificed and the implants directly explanted and preserved in 4% paraformaldehyde solution for further analyses (X‐ray microtomography, scanning electron microscopy, histology, and immunohistochemistry). All procedures involving live animals were approved by the McGill University Animal Ethics Committee following scientific review.

Decalcification of *N* = 3 explants per group was performed in ethylenediaminetetraacetic acid (EDTA, 14 wt%) at pH 7.2 for 3 weeks at 4 °C until the samples were radiolucent. Micro‐CT was performed at this stage on both calcified and decalcified samples. Dehydration in ascending serial ethanol solutions preceded PMMA (polymethyl methacrylate) embedding, followed by sectioning into 10 µm histological slices with a microtome (SP 1600 microtome Leica Microsystems, Germany). Hematoxylin and eosin staining, type‐IV collagen (Abcam ab6586), α‐smooth muscle actin (eBioscience 14‐9760‐82), and tartrate resistant alkaline phosphatase (Sigma‐Aldrich 387A‐1KT) stainings were performed. Histological imaging was performed using a Zeiss microscope Axio Imager.M2 (Zeiss Gottingen, Germany) with a digital AxioCam IC camera (Zeiss Gottingen, Germany). Scanning electron microscopy was performed on nonstained histological slides after platinum metallization, using back‐scattering electron mode.

CT‐Analyzer (Bruker) was used for the treatment of 3D µCT data and ImageJ (National Institutes of Health) for histological, immunohistological, and SEM analyses.

*Experiment Design and Surgical Procedures—Statistical Analysis*: Data are presented as representative images, representative experiments, or as means ± deviation, with *N* indicating the number of independent experiments. For SEM, three slices were measured per sample, with *N* = 4 for control and *N* = 5 for experimental groups. Means and standard deviations of each sample were combined using the formula

(1) $$\sigma =\sqrt{\frac{n_{1}\sigma_{1}^{2}+n_{2}\sigma_{2}^{2}+n_{1}{\left(\mu_{1}-\mu \right)}^{2}+n_{2}{\left(\mu_{2}-\mu \right)}^{2}}{n_{1}+n_{2}}}$$

using the Atozmath online calculator (http://atozmath.com/CONM/Ch2_CombinedSD.aspx).

One‐way analysis of variance (ANOVA) and Tukey posthoc test were performed using StatPage calculator (http://statpages.info/anova1sm.html).

## Conflict of Interest

The authors declare no conflict of interest.

## Supporting information

Supplementary
