# Supplementary material for: Material‐Induced Venosome‐Supported Bone Tubes
Source: Adv Sci (Weinh). 2019 Jul 1;6(17):1900844. doi: 10.1002/advs.201900844 (PMC6724474; doi:10.1002/advs.201900844)
Supplement: Supplementary file 1 — Supplementary [file ADVS-6-1900844-s001.pdf]

Copyright WILEY-VCH Verlag GmbH & Co. KGaA, 69469 Weinheim, Germany, 2019.

ADVANCED SCIENCE
Open Access

Supporting Information

for *Adv. Sci.,* DOI: 10.1002/advs.201900844

Material-Induced Venosome-Supported Bone Tubes

*Baptiste Charbonnier, Aslan Baradaran, Daisuke Sato, Osama Alghamdi, Zishuai Zhang, Yu-Ling Zhang, Uwe Gbureck, Mirko Gilardino, Edward Harvey, Nicholas Makhoul, and Jake Barralet**

Supporting Information

Title Material-Induced Venosome-Supported Bone Tubes

Author(s), and Corresponding Author(s)

Baptiste Charbonnier†, Aslan Baradaran†, Daisuke Sato, Osama Alghamdi, Zishuai Zhang, Yu Ling Zhang, Uwe Gbureck, Mirko Gliardino, Ed Harvey, Nicholas Makhoul†, Jake Barralet†

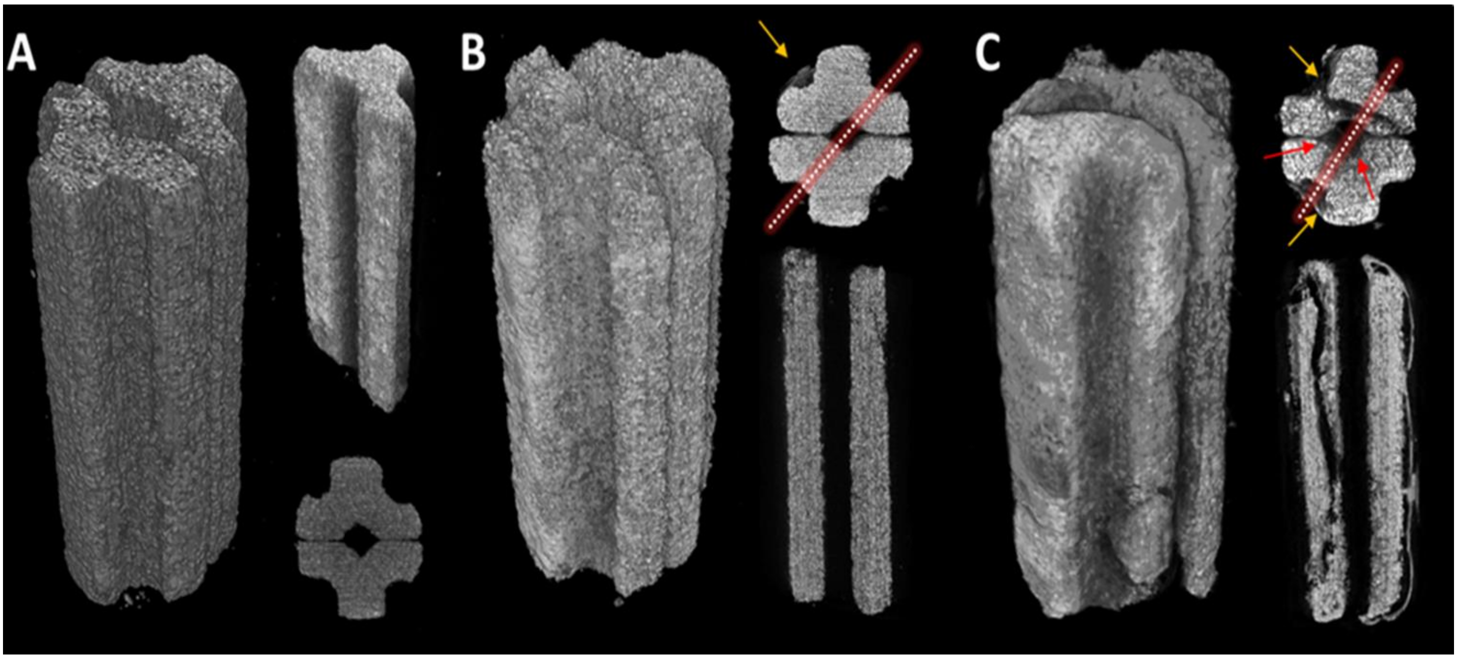

† Equal contributions

Figure S1. 3D reconstructions and representative sections of µCT of: A, Control prior to implantation, B, marrow loaded scaffold with no central vein, and C, venosome perfused marrow loaded scaffolds after 8 weeks subcutaneous implantation. In B and C an axial section is also shown along the plane indicated by the red dotted line. Bone was observed in the grooves where the marrow was loaded, (yellow arrows), that in the vascularised group (C) extended along the length of the scaffold. Bone was also observed inside the central channel (red arrows).

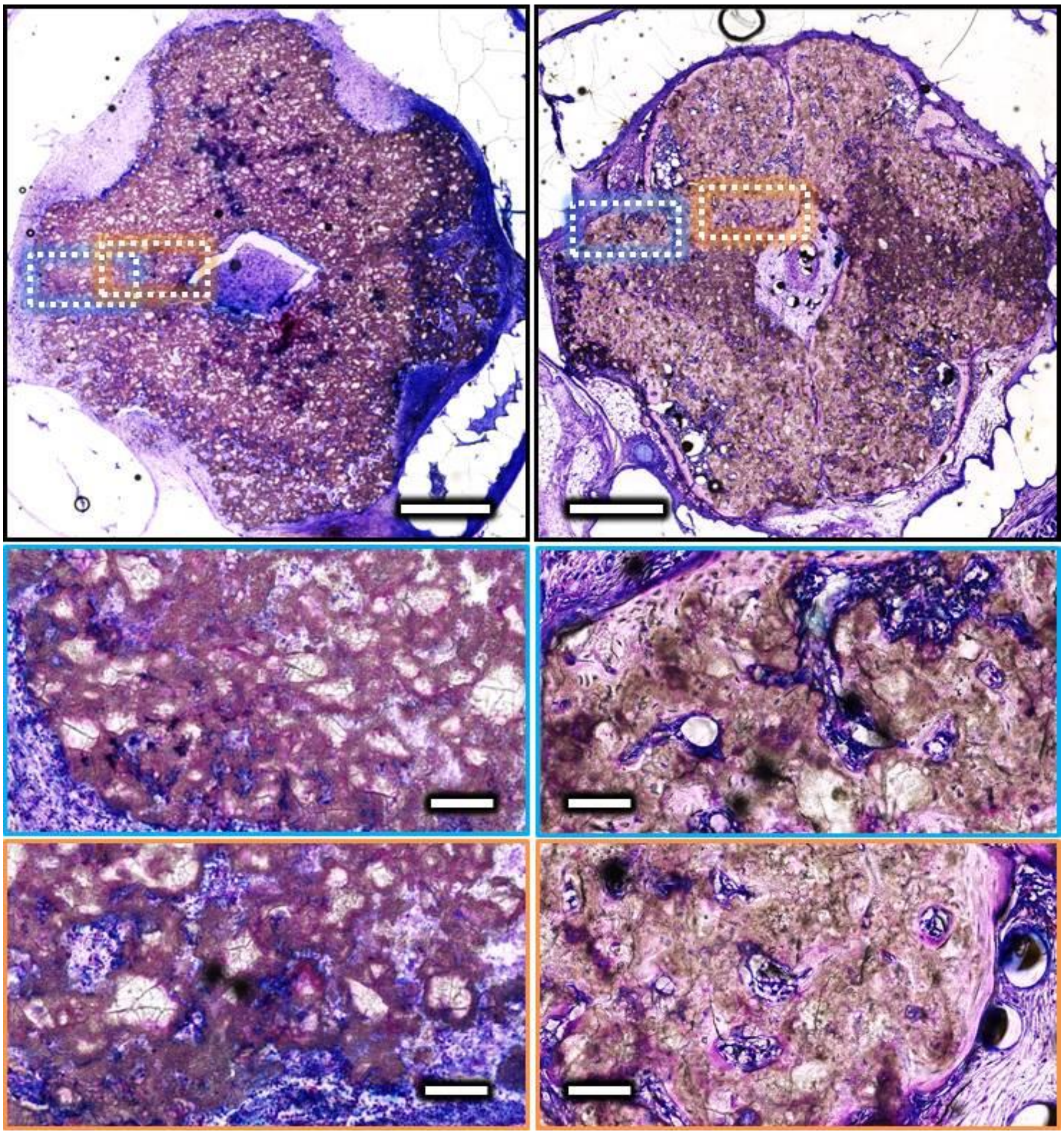

Figure S2. Mineralized axial section of the control (left) and the experimental (right) marrow constructs at low and high magnification, after basic fuchsin and methylene blue staining. Note relative absence of pink bone tissue with characteristic lacunae in non-vein perfused scaffold (left). Scale bar on low and high magnification represent 1 mm and 100 µm, respectively.
